# Privacy Protection and Secondary Use of Health Data: Strategies and Methods

**DOI:** 10.1155/2021/6967166

**Published:** 2021-10-07

**Authors:** Dingyi Xiang, Wei Cai

**Affiliations:** ^1^Internet Rule of Law Institute, East China University of Political Science and Law, Shanghai, China; ^2^Humanities and Law School, Northeast Forest University, Harbin, Heilongjiang, China; ^3^Beidahuang Information Company, Harbin, Heilongjiang, China

## Abstract

Health big data has already been the most important big data for its serious privacy disclosure concerns and huge potential value of secondary use. Measurements must be taken to balance and compromise both the two serious challenges. One holistic solution or strategy is regarded as the preferred direction, by which the risk of reidentification from records should be kept as low as possible and data be shared with the principle of minimum necessary. In this article, we present a comprehensive review about privacy protection of health data from four aspects: health data, related regulations, three strategies for data sharing, and three types of methods with progressive levels. Finally, we summarize this review and identify future research directions.

## 1. Introduction

The rapid development and application of multiple health information technologies enabled medical organizations to store, share, and analyze a large amount of personal medical/health and biomedical data, of which the majority are electronic health records (EHR) and genomic data. Meanwhile, the emerging technologies, such as smart phones and wearable devices, also enabled third-party firms to provide many kinds of complementary mHealth services and collect huge tons of consumer health data. Health big data has already been the most important big data for its serious privacy disclosure concerns and huge potential value of secondary use.

Health big data stimulated the development of personalized medicine or precision medicine. Empowered by health informatics and analytic techniques, secondary use of health data can support clinical decision making; extract knowledge about diseases, genetics, and medicine; improve patients' healthcare experiences; reduce healthcare costs; and support public health policies [1–3]. On the other side of the coin, health data contains much personal privacy and confidential information. For the guidance of protecting health-related privacy, the Health Insurance Portability and Accountability Act (HIPAA) of the US specifies 18 categories of protected health information (PHI) [4]. The heavy concerns about privacy disclosure much hinder secondary use of health big data. Much efforts tried to balance between privacy management and health data secondary use from both the legislation side [5] and the technology side [6, 7]. But for much more circumstances, a perfect balance is difficult to achieve; instead, a certain tradeoff or compromise must always be made. Recently, COVID-19 may perfectly illustrate the conundrum between protecting health information and ensuring its availability to meet the challenges posed by a significant global pandemic. In this ongoing battle, China and South Korea have mandated public use of contact tracing technologies, with few privacy controls; other countries are also adopting contact tracing technologies [7].

The direct and also important strategy to balance both issues is reusing health data under the premise of protecting privacy. The most primary idea is to share deidentified health data by removing 18 specified PHI. Based on deidentified health data, machine learning and data mining can be used for knowledge extraction or learning health system building for the purpose of analyzing and improving care, whereby treatment is tailored to the clinical or genetic features of the patient [8]. However, transforming data or anonymizing individuals may minimize the utility of the transferred data and lead to inaccurate knowledge [9]. This tradeoff between privacy and utility, also accuracy, is the center issue of sensitive data secondary usage [10]. Deidentification refers to a collection of techniques devised for removing or transforming identifiable information into nonidentifiable information and also introducing random noise into the dataset. By deidentification, privacy protection will be leveraged, but the outcome of analysis may be not exact, rather an approximation. To reconcile this conflict, the privacy loss parameter, also called privacy budget, was proposed to tune the tradeoff between privacy and accuracy: by changing the value of this parameter, more or less privacy resulting in less or more accuracy, respectively [11]. Furthermore, deidentified data may become reidentifiable through data triangulation from other datasets, which means that the privacy harms of big health data arise not merely in the collection of data but in their eventual use [12]. Just deidentification is far from needed. Instead, a holistic solution is the right direction, by which the risk of reidentification from records should be kept as low as possible and data be shared with the principle of minimum necessary [13]. For the minimum necessary, user-controlled access [6, 14] and secure network architecture [15] can be a practical implementation. For effective reusing health data while reducing the risk of reidentification, attempts in three aspects can be applicable references, that is, risk-mitigation methods, privacy-preserving data mining, and distributed data mining without sharing out data.

The remainder of this paper is organized as follows.  Section 2 describes the scope of health data and its corresponding category.  Section 3 summarizes regulations about privacy protection of health data in several countries.  Section 4 concisely reviews two strategies for privacy protection and secondary use of health data.  Section 5 reviews three aspects of tasks and methods for privacy preservation and data mining the primary tasks of data mining.  Section 6 concludes this study.

## 2. Health Data and Its Category

Generally speaking, any data associated with users' health conditions can be viewed as health data. The most important health data is clinical data, especially electronic medical records (EMR), produced by different level hospitals. With the development of health information technology and the popularization of wearable health device, vast amounts of health-relevant data, such as monitored physiological data and diet or exercise data, are collected from individuals and entities elsewhere, both passively and actively. According to the review article by Deven McGraw and Kenneth D. Mandl, health-relevant data can be classified into four categories [7]. In this research, we focus on the first two categories of data, which are directly related to users' health and privacy.

Category 1. Health data generated by healthcare system. This type of data is clinical data and is recorded by clinical professionals or medical equipment when a patient gets healthcare service in a hospital or clinic. Clinical data includes EMR, prescriptions, laboratory data, pathology images, radiography, and payor claims data. Patients' historical condition and current condition are recorded for treatment requirement. For making better health service for patients, it is important to track patients' lifelong clinical data and make clinical data sharing among different healthcare providers. Personal health record (PHR) was proposed to integrate patients' cross-institutions and lifelong clinical data [16]. This type of health data is generated and collected routinely in the process of healthcare, with the explicit aim that those data be used for the purpose of analyzing and improving care. For the purpose of clinical treatment, and also because of consumers' firm trust on healthcare experts and institutions, clinical data contains a high degree of health-related privacy. Therefore, the majority of health privacy laws mainly cover the privacy protection of clinical data [7]. Under the constraints of health privacy laws, tons of clinical data have been restricted only for internal use in medical institutions. Meanwhile, the clinical data is also extremely valuable for secondary usage since the data is created by professional experts and is direct description of consumers' health conditions. The tradeoff between utility and privacy of this type of health data has been one of the most important issues in the age of medical big data.

Category 2. Health data generated by consumer health and wellness industry. This type of health data is an important complementation to clinical data. With the widespread application of new-generation information technology, such as IoT, mHealth, smart phone, and wearable device, consumers' health attitude has greatly changed from passive treatment to active health. Consumers' health data can be generated through wearable fitness tracking devices, medical wearables such as insulin pumps and pacemakers, medical or health monitoring apps, and online health service. These health data can include breath, heart rate, blood pressure, blood glucose, walking, weight, diet preference, position, and online health consultation. These products or services and health data play important role in consumers' daily heath management, especially for chronic disease patients. This area has gained more and more focus from industry and academia. Consumer health informatics is the representative direction [17]. This type of nontraditional health-relevant data, often equally revealing of health status, is in widespread commercial use and, in the hands of commercial companies, yet often less accessible by providers, patients, and public health for improving individual and population health [18]. These big health data are scattered across institutions and intentionally isolated to protect patient privacy. For this type of health data, integration and linking at individual level are an extra challenge except for the utility-privacy tradeoff.

Table 1 summarizes the two categories of health data and their comparative features.

## 3. Regulations about Privacy Protection of Health Data

Personal information and health-relevant data are necessary to record in order to provide regular health service. Meanwhile, personal information and health-relevant data are closely associated with user privacy and confidential information. Therefore, several important privacy protection-related regulations or acts are published to guide health data protection and reuse. Modern data protection law is built on “fair information practice principles” (FIPPS) [19].

The most referenced regulation is Health Insurance Portability and Accountability Act (HIPAA) [4]. HIPAA was created primarily to modernize the flow of healthcare information, stipulate how personally identifiable information maintained by the healthcare and healthcare insurance industries should be protected from fraud and theft, and address limitations on healthcare insurance coverage. The HIPAA Safe Harbor (SH) rule specifies 18 categories of explicitly or potentially identifying attributes, called protected health information (PHI), that must be removed before the health data is released to a third party. HIPAA also covers electronic PHI, ePHI. This includes medical scans and electronic health records. A full list of PHI elements is provided in Table 2. PHI elements in Table 2 only cover identity information and do not include any sensitive attribute. That is, HIPAA does not provide guidelines on how to protect sensitive attribute data; instead, the basic idea of the HIPAA SH rule is to protect privacy by preventing identity disclosure. However, other sensitive attributes may still uniquely combine into a quasi-identifier (QI), which can allow data recipients to reidentify individuals to whom the data refer. Therefore, a strict implementation of the SH rule, however, may be inadequate for protecting privacy or preserving data quality. Recognizing this limitation, HIPAA also provides alternative guidelines that enable a statistical assessment of privacy disclosure risk to determine if the data are appropriate for release [20].

The Health Information Technology for Economic and Clinical Health (HITECH) Act [21] was enacted as part of the American Recovery and Reinvestment Act of 2009 to promote the adoption and meaningful use of health information technology. Subtitle D of the HITECH Act addresses the privacy and security concerns associated with the electronic transmission of health information, in part, through several provisions that strengthen the civil and criminal enforcement of the HIPAA rules. It is complimentary with HIPAA and strengthens HIPAA's privacy regulations. HITECH has also widened the scope of HIPAA through the Omnibus Rule. This extends the privacy and security reach of HIPAA/HITECH to business associates. According to HIPAA and HITECH Act, much of data beyond category 1 in Table 1 is outside of the scope of comprehensive health privacy laws in the U.S.

The Consumer Data Right (CDR) [22] is coregulated by the Office of the Australian Information Commissioner (OIAC) and Australian Competition and Consumer Commission (ACCC). “My Health Record System” is run to track citizen medical conditions, test results, and so on. The OIAC sets out controls on how health information in a My Health Record can be collected, used, and disclosed, which corresponds to PHR integration. The Personal Information Protection and Electronic Documents Act (PIPEDA) [23] of Canada applies to all personal health data. PIPEDA is stringent and although has many commonalities with HIPAA; it goes beyond HIPAA requirements in several areas. One such area is in the protection of data generated by mobile health apps which is not strictly covered by HIPAA. PIPEDA runs to protected consumer health data. Under PIPEDA, organizations can seek implied or explicit consent, which is based on the sensitivity of the personal information collected and the reasonable data processing consent expectations of the data subject. The General Data Protection Regulation (GDPR) is a wide-ranging data protection regulation in EU, which covering health data as well as all other personal data, even they contain sensitive attributes. GDPR also has data consent and breach notification expectations and contains several key provisions, including notification, right to access, right to be forgotten, and portability. Under GDPR, organizations are required to gain explicit consent from data subjects, and individuals have the right to restriction of processing and not to be subject to automated decision-making.

China has no specific regulations for health data privacy protection. Several restriction rules to prohibit privacy disclosure scatter in China Civil Code (CCC), Medical Practitioners Act of the PRC (MPAPRC), and Regulations on Medical Records Management in Medical Institutions (RMRMMMI), which make privacy disclosure restrictions to individuals, medical practitioners, and medical institutions, respectively. CCC specifies 9 categories of personal information to be protected, including name, birthday, ID number, biometric information, living address, phone number, email address, health condition information, and position tracking information. RMRMMMI only approves reuse of health data just for medical care, teaching, and academic research. Recently, the Personal Information Protection Law of the PRC (PIPILRC) [24] is released and will come into force on November 1, 2021. This is the first complete and comprehensive regulation on personal information protection. In this regulation, the definition of sensitive personal information and automatic decision making both involve health data, so, this regulation is applicable to privacy protection of health data. According to this regulation, secondary use of deidentified or anonymized health data for automatic decision making is permitted, and data processing consent from consumers is also required. This regulation, so far as can be foreseen, will greatly stimulate the exploitation and exploration of health big data.

According to the comparison of these data privacy relevant regulations, shown in Table 3, PIPEDA and GDPR and the newly released PIPILRC can cover both clinical data and consumer health data, and others pay the majority of attention to clinical data. Health data need to be reused for multiple important purposes. In fact, health data processing and reusing are never absolutely prohibited in the regulations mentioned above, as long as privacy protection is achieved as the important prerequisite. In this respect, HIPAA sets Safe Harbor rules to make sure PHI be removed before the health data is released to a third party. Furthermore, PIPEDA and GDPR require consumers' consent for data processing. Regulations from China also encourage health data to be reused in certain restricted areas. As the newcomer, PIPILRC presents a more complete and comprehensive guidance to protect and process health data.

## 4. Strategies and Framework

The exploitation of health data can provide tremendous benefits for clinical research, but methods to protect patient privacy while using these data have many challenges. Some of these challenges arise from a misunderstanding that the problem should be solved by a foolproof solution. There exists a paradox: well deidentified and scrubbed data may lose much meaningful information results in low quality, maintaining much PHI may have high risk of privacy breach. Therefore, a holistic solution, or to say a unified strategy, is needed. Three strategies are summarized in this section. The first is for clinical data and provides a practical user access rating system, and the second is majority for genomic data and designs a network architecture to address both security access and potential risk of privacy disclosure and reidentification. From a more practical starting point, the third tries to share a model without exposing any data. The tree strategies present solutions from different perspectives, therefore can be complementary to each other.

### 4.1. Strategies for Clinical Data

As for clinical data, Murphy et al. proposed an effective strategy to build a clinical data sharing platform while protecting patient privacy [6]. The proposed approach to resolving the balance between privacy management and data secondary use is to match the level of data deidentification with the trustworthiness of the data recipients, in which the more identified the data, the more “trustworthy” the recipients are required to be, and vice versa. The level of trust for a data recipient becomes a critical factor in determining what data may be seen by that person. This type of hierarchical access rating is similar to the film rating, which can accommodate the requirement and appetites of different types of audiences. Murphy et al.'s strategy sets up five patient privacy levels with three aspects of requirements: availability of the data, trust in the researcher and the research, and the security of the technical platforms. Corresponding to the privacy levels are five user role levels.

The lowest level of user is “obfuscated data user.” For this user, data are obfuscated as it is served to a client machine with possibly low technical security. Obfuscation methods try to add a random number to the aggregated counts instead of providing accurate result [25, 26]. The second level of user is “aggregated data user,” to whom exact numbers from aggregate query results are permissible. The third is “LDS data user,” who is granted to access HIPAA-defined LDS (limited dataset) and structured patient data in which PHI must be removed. The fourth is “Notes-enabled LDS data user,” who is additionally allowed to view PHI scrubbed text notes (such as discharge summary). The final level of user is “PHI-viewable data user,” who has access to all patient data.

These access level categories are summarized in Table 4.

With the guidance of health data access level categories, Murphy et al. implemented five cases in clinical research. In a realistic project, multiple use role or different access privileges must be needed to reconcile different data access requirements. Murphy et al. also provided three exemplar projects and their possible privacy level user distributions. This proposed strategy gave a complete reference for data sensitive project and also implemented a holistic approach to patient privacy solutions in Informatics for Integrating Biology and the Bedside (i2b2) research framework [27]. The i2b2 framework is the most widespread open-source framework for exploring clinical research data-warehouses and was jointly developed by the Harvard Medical School and Massachusetts Institute of Technology to enable clinical researchers to use existing deidentified clinical data and only IRB-approved genomic data for research aims. Yet, i2b2 does not provide any specific protection mechanism for genomic data.

### 4.2. Strategies for Genomic Data

**Figure 1:**
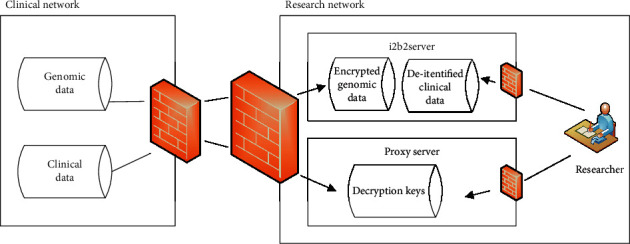
Network architecture of privacy protection for health data including genomic data.

As for genomic data, two potential privacy threats are loss of patients' health data confidentiality due to illegitimate data access and patients' reidentification and resulting sensitive attribute disclosure from legitimate data access. On the basis of the i2b2 framework, Raisaro et al. [15] proposed to apply homomorphic encryption [28] to the first threat and differential privacy [29] to the second threat. Furthermore, Raisaro et al. designed a system model, consisting of two physically separated networks, from the perspective of architecture. The network architecture is shown in Figure 1. This network architecture is aimed at isolating data that is used for clinical/medical care and that is used for research activities by a few trusted and authorized individuals.

The clinical network is used for hospital's clinical daily activities, containing clinical and genomic data of patients. This network is very controlled and protected by a firewall that blocks all incoming network traffic. Authorized users are permitted to log in.

The research network hosts i2b2 service used by researchers in their research activities. The i2b2 service is composed of an i2b2 server and a proxy server, in which a homomorphic encryption method and a differential privacy method are implemented and deployed. The i2b2 server can receive deidentified clinical data and encrypted genomic data from the clinical network and perform security data query and computation. The proxy server is devoted to support the decryption phase and the storage of partial decryption keys for homomorphic encryption. Through the research network, researchers can get authorized data via query execution module by the sequential five steps: query generation, query processing, result perturbation, result partial decryption, and result decryption at the final user-client side.

This network architecture and its privacy-preserving solution have been successfully deployed and tested in Lausanne University Hospital and used for exploring genomic cohorts in a real operational scenario. This application is also a practicable demonstration for similar scenario. It is not a unique instance but has its counterpart. Azencott reviewed how breaches in patient privacy can occur, and recent developments in computational data protection also proposed a similar secure framework for genomic data sharing around three aspects, which includes algorithmic solutions to deidentification, database security, and user trustworthy access [3].

### 4.3. Strategies for Sharing Not Data but Models

Since the new paradigm of the machine learning method, namely, federated learning (FL), was first introduced in 2016 [30], has achieved a rapid development, and become a hot research topic in the field of artificial intelligence, its core idea is to train machine learning models on separate datasets that are distributed across different devices or parties, which can preserve the local data privacy to a certain extent. This development mainly benefits from the following three facts [31]: (1) the wide successful applications of machine learning technologies, (2) the explosive growth of big data, and (3) the legal regulations for data privacy protection worldwide.

**Figure 2:**
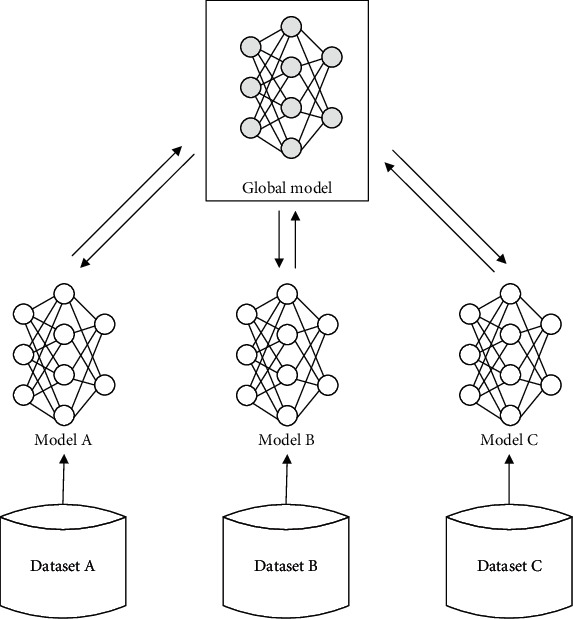
Architecture for a federated learning system.

The idea of federated learning is to only share the model parameters instead of the original data. By this way, many of these initiatives are based on federated models in which the actual data never leave the institution of origin, allowing researchers to share models without necessarily sharing patient data. Federated learning has inspired another important strategy to develop smart healthcare based on sensitive and private medical records which exist in isolated medical centers and hospitals. As shown in Figure 2, federated learning offers a framework to jointly train a global model using datasets stored in separate clients.

Model building of this kind has been used in real-world applications where user privacy is crucial, e.g., for hospital data or text predictions on mobile devices, and it has been stated that model updates are considered to contain less information than the original data, and through the aggregation of updates from multiple data points, original data is considered impossible to recover. Federated learning emphasizes the data privacy protection of the data owner during the model training process. Effective measures to protect data privacy can better cope with the increasingly stringent data privacy and data security regulatory environment in the future [32].

## 5. Tasks and Methods

Under the strategies of health data protection, specific tasks and methods about privacy and data processing can be employed and deployed. The tasks and methods can be viewed at three progressive levels. Methods in the first level are aimed at mitigating the risk of privacy disclosure, from four aspects. Methods in the second level target on data mining or knowledge extraction from deidentified or anonymized health data. No need to share health data, methods in the third level try to build a learning model or extract knowledge in a distributed manner, then share the model or knowledge.

### 5.1. Risk-Mitigation Methods

There are two widely recognized types of privacy disclosure [33]: identity disclosure (or reidentification) and attribute disclosure. The former occurs when illegitimate data users try to match a record in a dataset to an individual, and the latter occurs when illegitimate data users try to predict the sensitive value(s) of an individual record. According to Malin et al. [34], methods of mitigating the risk of two types of privacy disclosure can be divided into four classes: suppression, generalization, randomization, and synthetization. This perspective of method categories expects to well summarize the recent research on risk-mitigation methods.

#### 5.1.1. Suppression Methods

Suppression methods are aimed at scrubbing (remove or mask) 18 PHI defined in HIPAA, which is the most important deidentification method. Before PHI scrubbing, the major task is to identify the PHI from health data. For structural data, PHI identification can be done easily according to data schema. For narrative data or free text, such as discharge summary or progress note, natural language processing (NLP) is the preferred technology for PHI identification. Specifically, named entity recognition (NER) is the mainstream technology used in clinical data for deidentification and medical knowledge extraction. The 18 PHI are regarded as predefined entity types, and machine learning is employed to annotate type tags for each word in a sentence, then those tags are merged, and finally, the position and type of PHI can be identified. Conditional random fields (CRFs) are the classic sequential tagging model for NER and are often applied for deidentification [35]. Meystre et al. made a systematic review of deidentification methods [36], and Uzuner et al. [37] and Deleger et al. [38] both conducted some evaluations on a certain human-annotated dataset. The identified PHI values are then simply removed from or replaced with a constant value in the released text documents, which may be inadequate for protecting privacy or preserving data quality. Li and Qin proposed a new systematic approach to integrate methods developed in both data privacy and health informatics fields. The key novel elements of the proposed approach include a recursive partitioning method to cluster medical text records and a value enumeration method to anonymize potentially identifying information in the text data, which essentially masks the original values, to improve privacy protection and data utility [20].

For genomic data, homomorphic encryption [28] is applied to encrypting genomic data, and then, encrypted data can be shared for secondary use. Raisaro et al. employed homomorphic encryption to build a data warehouse for genomic data [15]. Kamm et al. [39] also proposed a framework for generating aggregated statistics on genomic data by using secure multiparty computation based on homomorphic secret sharing. Several other works [28, 40, 41] proposed using homomorphic encryption to protect genomic information in order to allow researchers to perform some statistics directly on the encrypted data and decrypt only the final result.

#### 5.1.2. Generalization Methods

These methods transform data into more abstract representations. The much easier implementation is abbreviation. For instance, the age of a patient may be generalized from 1-year to 5-year age groups. Based on this type of generation, sensitive attributes can be generalized subgroup and be anonymized to some extent, which is the back idea of *k*-anonymity and its variations. *k*-anonymity seeks to prevent reidentification by stripping enough information from the released data that any individual record becomes indistinguishable from at least (*k* − 1) other records [42]. The idea of *k*-anonymity is based on modifying the values of the QI attributes to make it difficult for an attacker to unravel the identity of persons in a particular dataset while the released data remain as useful as possible. This modification is a sort of generalization, by which stored values can be replaced with semantically consistent but less precise alternatives [43]. For example, let us consider a dataset in which age is a quasi-identifier. While the three records {age = 30, gender = male}, {age = 35, gender = male}, and {age = 31, gender = female} are all distinct, releasing them as {age = 3∗, gender = male}, {age = 3∗, gender = male}, and {age = 3∗, gender = female} ensures they all belong to the same age category and the anonymity is 3-anonymity. Based on *k*-anonymity, *l*-diversity [44, 45] were proposed to address further disclosure issues of sensitive attributes.

#### 5.1.3. Randomization Methods

Randomization can be used for attribute-level data. In this case, original sensitive values are replaced with similar but different values, with a certain probability. For example, a patient's name may be masked by a randomly selected made-up name. This basic approach may result in worse data quality. Li and Qin proposed to obtain value via a clustering method [20].

Randomization can further be used for aggregation operation. Obfuscation is a sort of such randomization. Numerous repetitions of a query by a single user must be detected and interrupted because they will converge on the true patient count making proper user identification absolutely necessary for the methods to function properly [6]. Aiming to deidentify aggregated data, obfuscation methods include the addition of a random number to the patient counts that has a distribution defined by a Gaussian function.18. Obfuscation is applied to aggregate patient counts that are reported as a result of ad hoc queries on the client machine [26]. Another protection model for preventing reidentification is differential privacy [10, 46]. In this model, reidentification is prevented by the addition of noise to the data. The model is based on the fact that auxiliary information will always make it easier to identify an individual in a dataset, even if anonymized. Instead, differential privacy seeks to guarantee that the information that is released when querying a dataset is nearly the same whether a specific person is included or not [46]. Unlike other methods, differential privacy provides formal statistical privacy guarantees.

#### 5.1.4. Synthetization Methods

Synthetization is compelling for two main reasons: preserving confidentiality and valid inferences for various estimates [47]. In this case, the original data are never shared. Instead, general aggregate statistics about the data are computed, and new synthetic records are generated from the statistics to create fake, but realistic-like, data. Exploiting clinical data for building an intelligent system is one of the scenarios. Developing clinical natural language processing systems often requires access to many clinical documents, which are not widely available to the public due to privacy and security concerns. To address this challenge, Li et al. proposed to develop methods to generate synthetic clinical notes and evaluate their utility in real clinical natural language processing tasks. Thanks to the development of deep learning, recent advances in text generation have made it possible to generate synthetic clinical notes that could be useful for training NER models for information extraction from natural clinical notes, thus lowering the privacy concern and increasing data availability [48].

### 5.2. Privacy-Preserving Data Mining

Data mining is also synonymously called knowledge discovery from data (KDD), which highlights the goal of the mining process. To obtain useful knowledge from data, the mining process can be divided into four iterative steps: data preprocessing, data transformation, data mining, and pattern evaluation and presentation. Based on the stage division in the process of KDD, Xu et al. developed a user-role-based methodology and identified four different types of users in a typical data mining scenario: data provider, data collector, data miner, and decision maker. By differentiating the four different user roles, privacy-preserving data mining (PPDM) can be explored in a principled way, by which all users care about the security of sensitive information but each user role views the security issue from its own perspective [49]. In this research, PPDM is explored from the view of a data miner role, that is, from the data mining stage of KDD.

Privacy-preserving data mining is aimed at mining or extracting information, via a certain machine learning-based model, from privacy-preserving data in which the values of individual records have been perturbed or masked [50]. The key challenge is that the privacy-preserving data look very different from the original records and the distribution of data values is also very different from the original distribution. Researches for this issue have started very early. Agrawal and Srikant proposed a reconstruction procedure to estimate the distribution of original data values and then built a decision-tree classifier [50]. Recent studies on PPDM include privacy-preserving association rule mining, privacy-preserving classification, and privacy-preserving cluster.

Association rule mining is aimed at finding interesting associations and correlation relationships among large sets of data items. For PPDM, some of the rules may be considered to be sensitive. For hiding these rules, the original data need to be modified to generate a sanitized dataset from which sensitive rules cannot be mined, while those nonsensitive ones can still be discovered [51]. Classification is a task of data analysis that learns models to automatically classify data into defined categories. Privacy-preserving classification evolves decision tree, Bayesian model, support vector machine, and neural classification. The strategies of adapting the classification method to a privacy-preserving scenario can simply be described as two aspects. The first is learning the classification model based on data transformation, since the transformed data is difficult to be recovered [52, 53]. The second is learning the classification model based on secure multiparty computation (SMC) [54], where multiparties collaborate to develop a classification model from vertically partitioned or horizontally partitioned data, but no one wants to disclose its data to others [55, 56]. Cluster analysis is the process of grouping a set of records into multiple groups or clusters so that objects within a cluster have high similarity but are very dissimilar to objects in other clusters. This process runs in an unsupervised manner. Similar to classification, current researches on privacy-preserving clustering can be roughly categorized into two types, based on data transformation [57, 58] and based on secure multiparty computation [59, 60].

### 5.3. Federated Privacy-Preserving Data Mining

For the distributed or isolated data, distributed data mining is the research topic. Distributed data mining can be further categorized into data mining over horizontally partitioned data and data mining over vertically partitioned data. Research on distributed data mining attracts much attention. To overcome the difficulty of data integration and promote efficient information exchange without sharing sensitive raw data, Que et al. developed a Distributed Privacy-Preserving Support Vector Machine (DPP-SVM). The DPP-SVM enables privacy-preserving collaborative learning, in which a trusted server integrates “privacy-insensitive” intermediary results [61]. In medical domain, much raw data can hardly leave the institution of origin. Instead of bringing data to a central repository for computation, Wu et al. proposed a new algorithm, Grid Binary LOgistic REgression (GLORE), to fit a LR model in a distributed fashion using information from locally hosted databases containing different observations that share the same attributes [62].

It is worth to note that learning (classification or clustering) on secure multiparty computation is an important distributed learning strategy, by which privacy disclosure concern can be much reduced since data need not to be shared out. This research topic probably inspired federated machine learning [30, 32]. Today's AI still faces two major challenges. One is that data exists in the form of isolated islands. The other is the strengthening of data privacy and security. The two challenge is much severer in the healthcare domain. Federated machine learning is aimed at building a learning model from decentralized data [30]. Federated learning can be classified into horizontally federated learning, vertically federated learning, and federated transfer learning based on how data is distributed among various parties in the feature and sample ID space [32]. Horizontal federated learning, or sample-based federated learning, is introduced in the scenarios that datasets share the same feature space but different in samples. At the end of the learning, the universal model and the entire model parameters are exposed to all participants. Vertical federated learning or feature-based federated learning is applicable to the cases that two datasets share the same sample ID space but differ in feature space. At the end of learning, each party only holds the model parameters associated with its own features; therefore, at inference time, the two parties also need to collaborate to generate output. Federated transfer learning (FTL) applies to the scenarios that the two datasets differ not only in samples but also in feature space. FTL is an important extension to the existing federated learning systems and is more similar to vertical federated learning. The challenge of protecting data privacy while maintaining the data utility through machine learning still remains. For a comprehensive introduction of federated privacy-preserving data mining, please refer to the survey based on the proposed 5 W-scenario-based taxonomy [31].

### 5.4. Summary: Privacy vs. Accuracy

Privacy protection is the indispensable prerequisite of secondary usage of health data. As discussed above, risk-mitigation methods are aimed at anonymizing private or sensitive information so as to reduce the risk of reidentification. Methods about privacy-preserving data mining target to process the privacy-scrubbed data and extract knowledge and even build AI systems. If absolute privacy safe is pursued, the scrubbed data is definitely useless, since the data quality is severely corrupted. With the poor-quality data, accuracy and effectiveness of data utilization are extremely affected. Therefore, in a practical scenario, a certain tradeoff or compromise between privacy and accuracy must always be made. The tradeoff can be tuned to provide more or less privacy resulting in less or more accuracy, respectively, according to the requirements of privacy level and utility level. Federated privacy-preserving data mining sheds light on the new direction to compromise, even to balance, the privacy and accuracy. No need to share data out, federated privacy-preserving data mining first processes the original health data within institutions, and the conduct federated mining or learning. This type of method is expected to reconcile privacy and accuracy with more elegant style and more acceptable way.

## 6. Conclusions

Clinical data, genomic data, and consumer health data are the majority of health big data. Protection and reuse always gain much focused research topics. In this review article, the type and scope of health data are firstly discussed, followed by the related regulations for privacy protection. Then, strategies for user-controlled access and secure network architecture are presented. Sharing trained model without original data leaving out is a new important strategy and gains more and more focus. According to different data reuse scenarios, tasks and methods at three different levels are summarized. The strategies and methods can be combined to form a holistic solution.

With the rapid develop health information technology and artificial intelligence, the capability of privacy protection will impede the urgent demand of reusing health data. Some potential research directions may include (1) applying modern machine learning to deidentification and anonymization for multimodal health data while ensuring its data quality; (2) learning model construction and knowledge extraction based on anonymized data to leverage secondary use of health data; (3) federated learning on isolated heath data can both protect privacy perfectly and improve the efficiency of data transferring and processing, being deserved more attention; (4) research on alleviating reidentification risk, such as linkage or inference, from a trained model.

## Figures and Tables

**Table 1:** Summarization of clinical data and consumer health data.

	Category 1: clinical data	Category 2: consumer health data
Generated/record by	Healthcare system Clinical professionals Medical equipment	Wearable device (wristband, watch) Medical wearable Health App
Data detail	Name, id, age, address, phone, medical history, family history, conditions, laboratory test, treatments, prescriptions, etc.	Name, id, phone, address, position, age, weight, heart rate, breath, blood pressure, blood glucose, exercise data, diet preference, online health consultation, etc.
Data characteristics	Discrete but more professional, more clinical information and more privacy, stored in healthcare system, passive	Continuous but less standardization, more health information, privacy tend to be ignored, stored by different providers, active, vast amounts

**Table 2:** Protected health information defined by HIPAA.

Category	Description
1	Names
2	Locations
3	Dates
4	Phone number
5	Fax numbers
6	E-mail addresses
7	Social security numbers
8	Medical record numbers
9	Health plan beneficiary numbers
10	Account numbers
11	Certificate/license numbers
12	Vehicle identifiers and serial numbers
13	Device identifiers and serial numbers
14	Web Universal Resource Locators (URLs)
15	Internet Protocol (IP) address numbers
16	Biometric identifiers, including finger and voice prints
17	Full face photographic images and any comparable images
18	Any other unique identifying number, characteristics, or code

**Table 3:** Regulations and corresponding data category.

Regulations	Category 1: clinical data	Category 2: consumer health data
HIPAA & HITECH (USA)	✓	
CDR (Australia)	✓	
PIPEDA (Canada)	✓	✓
GDPR (EU)	✓	✓
MPAPRC & RMRMMMI (China)	✓	
CCC & PIPILRC (China)	✓	✓

**Table 4:** Health data access level categories.

Privacy level of user	Data available	Trustworthiness of user	Technical security
Obfuscated data user	Users have access to data by client-side application only	Low: only obfuscated aggregate results are available	Low: only client-side application exposed to users
Aggregated data user	Users have access to HIPAA deidentified data by client-side application only	Low: users can get exact patient counts against deidentified data	Low: but data manager assumes burden of deidentifying data
LDS data user	HIPAA-defined LDS and deidentified structured data	Medium: users can see LDS as defined by HIPAA	Medium: requires user-facing direct access to the database
Notes-enabled LDS data user	HIPAA deidentified data and deidentified narrative text	Medium: users see both LDS and narrative text that is mostly deidentified	Medium: requires user-facing direct access to the database
PHI-viewable data user	All patient data may be accessed	High: users can see all protected health information on patients	High: requires management of encryption keys
